# Intestinal Alterations, Basal Hematology, and Biochemical Parameters in Adolescent Rats Fed Different Sources of Dietary Copper

**DOI:** 10.1007/s12011-015-0522-1

**Published:** 2015-10-02

**Authors:** Ewa Tomaszewska, Piotr Dobrowolski, Małgorzata Kwiecień

**Affiliations:** Department of Animal Physiology, Faculty of Veterinary Medicine, University of Life Sciences in Lublin, 12 Akademicka Street, 20-950 Lublin, Poland; Department of Comparative Anatomy and Anthropology, Maria Curie-Skłodowska University, Lublin, Poland; Institute of Animal Nutrition and Bromatology, University of Life Sciences in Lublin, Lublin, Poland

**Keywords:** Copper, Jejunum histomorphometry, Blood biochemistry, Rat

## Abstract

Copper (Cu) is required for basically all biochemical and physiological processes in the body. The aim was to evaluate the effects of different sources of dietary copper on jejunal epithelium histomorphometry in adolescent rats. Male rats at the age of 5 weeks were used in the 12-week experiment. The control group was fed with standard diet providing the required Cu level (5 mg/kg body weight (bw) per day) in an inorganic form (sulfate) covered 100 % of daily demand, and the other three groups were supplemented with Cu-glycine complex covered 50, 75, and 100 % daily demand. Basal hematological and plasma biochemical analyses as well as histomorphometric examinations of the jejunal epithelium and liver were performed. Cu given in the organic form in 100 % of daily demand depressed the muscular and submucosa layer and the crypt depth (*P* < 0.05) without an influence of the innervation of the jejunum. In turn, organic Cu given in 75 % of daily demand did not influence the intestinal morphology in adult rats. Dietary organic Cu given to rats covering the daily demand in 50 or 75 % appears to be less harmful with regard to the intestinal epithelium than when administered in 100 % of daily demand.

## Introduction

It is well known that trace minerals such as copper (Cu) are required for normal functioning of basically all biochemical and physiological processes in the body. It is one of the most abundant essential trace minerals after iron and zinc. The average content of Cu in tissues of growing mammals is three times higher than in adults [1, 2]. The required amount and tissue content of Cu in the body alters throughout the lifetime in animals and humans. The liver and kidney contain the highest amount of Cu among all tissues [1, 2]. Copper as a trace element is needed to maintain optimal body function, growth, reproduction, and immune response, determining in general the health status [3].

The imbalance of Cu homeostasis causes different health problems. The deficiency of Cu leads to anemia and bone malformation, problems with locomotor function, and increases the risk of developing osteoporosis in later life [3, 4]. Moreover, its deficit causes alterations in cholesterol and glucose metabolism with a resulting increase in the glucose and cholesterol level [3, 4]. On the other hand, Cu excess is toxic and generates free radicals [5]. Elevated Cu concentration can cause toxicosis and degeneration of liver tissue as well as brain damage like in Wilson’s disease [3, 6].

Cu absorption in the amount of 50 to 80 % of ingested Cu takes place in the small intestine [3]. Moreover, this absorption is influenced by iron, manganese, zinc, and cobalt [3]. Additionally, the chemical form in which the microelement is present changes its bioavailability [7–9]. Copper in animal and human diet can be given in inorganic sources (as copper sulfate or carbonate) or organic sources as amino acid chelate with higher Cu bioavailability due to the fact that additional connection with amino acid is not required at the brush border and the membrane transport is more rapid [3, 7, 10, 11].

Animal studies show that reduction of the concentrations of Cu typically supplemented to pig diets greatly decreases fecal mineral excretion without negatively affecting pig performance from weaning through development [12]. This is in agreement with a study where the use of lower levels of diet minerals caused a 21 % reduction in excretion of Cu [13]. It seems that farm animals are overfed trace minerals, and the use of a mineral chelate allows maintenance of performance, concomitantly reducing the mineral content in manure [13, 14]. The cause of the reduction in the level of trace minerals in the organic form could be related to the fact that rats administered with Cu chelate in 100 % of daily demand showed considerable damage to liver (large increase in apoptotic cells, ballooning degeneration, and vacuolization) compared to diets containing the same levels of Cu provided from sulfate [15]. Despite the fact that the role of Cu in animal and human health is well established, there is no knowledge about the influence of different sources thereof in the diet on the intestinal epithelium.

Dietary trace mineral sources and levels were administered to adolescent rats to evaluate their developmental responses during the growth phase on the basis of the histomorphometric analysis of the jejunal epithelium.

## Materials and Methods

The experimental procedures used throughout this study were approved by the Local Ethics Committee on Animal Experimentation of University of Life Sciences of Lublin, Poland. The rats were maintained in an animal house according to the guidelines of this committee. Experiment complied with the Guiding Principles for Research Involving Animals.

### Animal, Breeding, and Experimental Design

Male Wistar rats (*n* = 48) at the age of 5 weeks at the start of the experiment were used in the experiment lasted 12 weeks. Clinically healthy rats were individually kept in Macrolon cages (Bioscape, Emmendingen, Germany) at 21 ± 1 °C and 55 % humidity and 12-h light and dark cycles. All animals had free access to water and fed ad libitum. Adolescent rats were randomly divided into the control group (*n* = 12), in which Cu was provided by conventional inorganic salts (sulfate) and three experimental groups (each *n* = 12) fed different level of the organic Cu as Cu-glycine complex. The control group was fed with standard diet provided Cu in 100 % of daily demand from sulfate (5 mg/kg body weight (bw) per day; LSM, Agropol S.J., Motycz, Poland). The composition of basal diet is crude protein min. 14.5 %, crude fat min. 1.5 %, ash 10 %, and crude fiber min. 5 %. The content of vitamin and mineral premixes of the diet is presented in Table 1. On the other hand, experimental animals were fed with the same standard diet, but the experimental treatment contained 0 % of Cu in the diet. Copper was given to distilled water as Cu amino acid chelate (Cu-glycine complex) in the amount of 0.025 mg Cu/L H_2_O in the OG100 group (covered 100 % of daily demand), in the amount of 0.01875 mg Cu/L H_2_O in the OG75 group (covered 75 % of daily demand) and in the amount of 0.0125 mg Cu/L H_2_O in the OG50 group (covered 50 % of daily demand). The Cu-glycine complex covering 100 % of daily demand and the inorganic salts (sulfate) contained an equivalent mineral concentration. Water consumption during 24 h for the four groups of rats was measured before the beginning of the experiment, and the data were used to calculate the needed amount of Cu. These data, combined with body weight, Cu content in the chelate, and the amount of the chelate covering Cu demand, were used to calculate the amount of Cu at the dose of 5 mg/kg bw/day in distilled water. At the end of experiment, rats were fasted for 24 h and euthanized one by one with carbon dioxide inhalation and by dislocation of the spine.

**Table 1:** The composition of vitamin and mineral premixes of the diet (per kilogram dry matter) fed to rats during the study

Components	Per 1 kg of premix
Manganese, mg	5000
Iron, mg	5000
Zinc, mg	2500
Jodine, mg	75
Pantothenic acid (D-calcium pantothenate), mg	900
Retinol acetate (UI)	800,000
Cholecalciferol (UI)	100,000
Vitamine E, mg	4964
Menadione sodium bisulfite, mg	300
Riboflavin, mg	600
Pyridoxine HCL, mg	60
Cyanocobalamin, mg	1.2

### Hematological and Plasma Biochemical Analyses

Blood samples were collected carefully for hematological and blood plasma biochemical analyses by the heart venipuncture. Hematological analyses were performed with the use of an automatic hematological analyzer MS9 (Melet Schloesing Laboratories, France). The numbers of white and red blood cells (WBC and RBC), hemoglobin concentration (Hb), and hematocrit (HT) were determined.

The plasma was immediately separated by centrifugation and stored at −25 °C for further analysis. The plasma concentration of Cu, Fe, and Zn was determined by the colorimetric method using a Metrolab 2300 GL unit (Metrolab SA, Buenos Aires, Argentina) and ready-made sets produced by the company BioMaxima (Lublin, Poland).

Total protein, glucose, total cholesterol (TC), triacylglicerol (TG), low-density lipoprotein cholesterol (LDL-C), high-density lipoprotein cholesterol (HDL-C), lactate dehydrogenase (LDH), and alkaline phosphatase (ALP) were determined by the colorimetric method using a Metrolab 2300GL random access biochemical analyzer (Metrolab SA, Buenos Aires, Argentina) and tests by BioMaxima (Lublin, Poland).

### Tissue Collection and Histomorphometrical Analysis

A 15-mm-long segment from the same point in 50 % of the total jejunum length was taken from each animal of each group and was subjected to histology. Briefly, these samples of small intestine were opened along the mesenteric border, placed flat without stretching in standard histopathological cassettes (Bio-Optica Milano S.p.A, Mediolan, Italy) and fixed in 4 % buffered formaldehyde (pH 7.0) for 24 h, then dehydrated in graded series of ethanol and embedded in paraffin. Twenty cross sections, 4 μm thick, were cut with a microtome (Microm HM 360, Microm, Walldorf, Germany) with 20 μm interval after each five slices and were placed on poly-L-lysine-coated slides (Menzel Glasser, Braunschweig, Germany) and then stained using Masson’s trichrome method to differentiate the small intestine wall layers [16]. Microscopic images were collected using a microscope (Axiovert 200 M, Carl Zeiss, Jena, Germany). Objective magnifications of 4×, 10×, 20×, and 40× were used to show the different intestinal structures and to collect images of the examined tissues from each specimen for further analysis. The structure of the small intestine wall was examined under microscopic observation and with the use of graphic analysis software Olympus cellSens Version 1.5 (Olympus, Tokyo, Japan).

The following morphometric variables in the intestine were analyzed: mucosa, submucosa, and myenteron (longitudinal and transversal lamina) thickness (30 measurements, with the use of straight line, from the bottom to the top of each layer, of every sample); villar epithelium thickness (with the use of straight line, from the bottom to the top of epithelium cells, from 30 villi of each animal); enterocyte number per 100 μm of the villus (from 30 villi of each animal); crypt depth (defined as the depth of the invagination between adjacent villi from the bottom of the crypt to the base of villi); crypt width (measured in the middle of the crypt depth); the number of crypts (active: showing mitoses and Paneth cells, having an open internal space and access to the intestinal lumen; inactive: showing no mitoses and Paneth cells, having a closed internal space; total: active plus inactive crypts); villar length (from the tip of the villi to the villous-crypt junction); villar thickness (measured in the middle of villar height); the number of villi (100 well-defined villi from each animal); and small intestine absorptive surface as described previously [17].

## Statistical Analysis

All the results are expressed as means ± standard deviation (SD). Differences between the means were tested with the one-way ANOVA and post hoc Tukey’s test as the correction for multiple comparisons. Normal distribution of data was examined using the W. Shapiro-Wilk test, and equality of variance was tested by the Brown-Forsythe test. A *P* value of less than 0.05 was considered statistically significant. All statistical analyses were carried out by means of Statistica 12 software (StatSoft, Inc., Tulsa, OK, USA; http://www.statsoft.com).

## Results

### Food Consumption and Body Mass

Food consumption was measured daily in control animals and treated with Cu, and there was no differences (data not shown).

The average initial body mass of all the rats reached the value of 225 ± 30 g. The final body mass of the control rats and animals treated with the organic Cu source (regardless of the amount of daily demand) was similar and reached the value of 405.0 ± 50.0, 406.6 ± 39.0, 419.0 ± 50.0, and 411.9 ± 42.4 g in the control, OG100, OG75, and OG50 group, respectively.

### The Content of Cu in Blood Plasma

The Cu plasma concentration of the control rats and animals supplemented with the organic form of Cu (irrespective of the amount of Cu in the diet) did not differ between the groups (Table 2).

**Table 2:** Basal hematology and blood plasma biochemical parameters of control rats and supplemented with different level of dietary Cu

	CONT	OG100	OG75	OG50
WBC [10^9^/L]	5.77 ± 0.91	6.11 ± 1.47	4.43 ± 1.37	5.33 ± 1.05
RBC [10^12^/L]	7.96 ± 0.87	8.81 ± 0.52	8.92 ± 0.28	8.44 ± 0.17
Hb [mmol/L]	9.43 ± 1.01	9.79 ± 0.26	9.56 ± 0.32	9.45 ± 0.44
Ht	0.41 ± 0.02	0.42 ± 0.01	0.41 ± 0.02	0.41 ± 0.03
Total protein [g/L]	70.66 ± 4.23	67.14 ± 2.79	70.71 ± 3.3	71.66 ± 2.06
Glucose [mmol/L]	7.47 ± 0.48^a^	10.21 ± 1.92^b^	11.18 ± 4.01^a^	11.85 ± 4.81^a^
TC [mmol/L]	2.12 ± 0.06	2.09 ± 0.19	2.31 ± 0.2	2.27 ± 0.07
LDL-C [mmol/L]	0.37 ± 0.03	0.39 ± 0.07	0.41 ± 0.08	0.39 ± 0.01
HDL-C [mmol/L]	1.46 ± 0.06	1.36 ± 0.19	1.55 ± 0.25	1.54 ± 0.14
TG [mmol/L]	1.54 ± 0.24	1.75 ± 0.62	1.79 ± 0.26	1.72 ± 0.66
Cu [μmol/L]	27.69 ± 4.65	25.82 ± 1.71	28.38 ± 1.17	29.37 ± 4.54
Fe [μmol/L]	43.61 ± 1.86	40.76 ± 2.99	39.35 ± 2.83	42.01 ± 5.46
Zn [μmol/L]	27.65 ± 3.22	29.04 ± 3.73	29.87 ± 6.18	29.34 ± 4.42

Data given are mean ± SD

*CONT* the control group received Cu in 100 % of daily demand from sulfate, *OG100* the group received Cu in 100 % of daily demand from Cu glycine, *OG75* the group received Cu in 75 % of daily demand from Cu glycine, OG50 the group received Cu in 50 % of daily demand from Cu glycine

Differences between superscript letters means significant differences with *P* < 0.05

### Blood Hematology and Blood Plasma Biochemistry

The basal blood hematology in the control group did not differ from the value obtained in the groups treated with Cu-glycine complex in 100, 75, or 50 % of daily demand except that the number of white blood corpus was decreased in the OG75 group (Table 2). There was no difference in basal biochemical parameters between the control group and rats treated with the organic Cu (independently on the amount of daily demand), except glucose concentration, which significantly increased in the group fed with the organic form in 100 % of daily demand (Table 2).

### Gastrointestinal Track Morphology

The intake of Cu in Cu-glycine complex at the level of 100 % of daily demand resulted in reduction of the thickness of the villar epithelium compared to the control group (supplemented with Cu in the inorganic form at the level of 100 % of daily demand) and the group supplemented with the Cu amino acid chelate in 75 and 50 % of daily demand (Table 3). The number of enterocytes also decreased in this group compared to the control group (supplemented with Cu in the inorganic form at the level of 100 % of daily demand) and the group supplemented with the Cu amino acid chelate in 75 % of daily demand (Table 3).

**Table 3:** The histomorphometry of jejunal epithelium of control rats and supplemented with different level of dietary Cu

	CONT	OG100	OG75	OG50
Myenteron thickness [μm]:				
Longitudinal lamina	25.2 ± 6.9	23.6 ± 6.5	19.4 ± 8.3	27.2 ± 6.7
Transversal lamina	36.2 ± 9.3	28.1 ± 8.9	30.1 ± 8.3	34.0 ± 6.7
Submucosa thickness [μm]	31.7 ± 9.4	20.9 ± 6.3	26.5 ± 8.5	39.8 ± 6.9
Mucosa thickness [μm]	606.3 ± 111.8	475.2 ± 95.5	468.4 ± 82.6	649.1 ± 62.3
Enterocyte number/100 μm of villus	20.8 ± 2.9^a^	16.9 ± 1.9^b^	21.1 ± 3.4^a^	18.7 ± 2.9^a^
Villi epithelium thickness [μm]	30.0 ± 2.8^a^	23.7 ± 3.4^b^	35.5 ± 6.4^a^	31.1 ± 7.3^ab^
Villi length [μm]	588.4 ± 125.6	453.9 ± 90.9	445.8 ± 74.4	617.0 ± 55.5
Villi thickness [μm]	86.7 ± 17.4	83.3 ± 10.2	102.8 ± 20.9	112.1 ± 17.3
Total villi number/mm	8.7 ± 1.8	6.3 ± 2.8	6.4 ± 2.7	6.8 ± 1.6
Crypts depth [μm]	124.4 ± 32.8	105.0 ± 19.2	137.7 ± 28.9	122.4 ± 21.5
Crypts width [μm]	38.6 ± 9.2	34.7 ± 6.8	41.7 ± 6.4	34.7 ± 7.3
Active crypt number/mm	8.1 ± 2.8	6.2 ± 2.4	7.8 ± 3.1	7.3 ± 3.7
Inactive crypt number/mm	8.0 ± 3.7	11.1 ± 3.4	10.1 ± 2.8	8.0 ± 3.0
Total crypt number/mm	15.9 ± 3.9	17.3 ± 3.2	17.9 ± 2.5	15.3 ± 2.2
Small intestine absorptive surface [μm^2^]	11.1 ± 3.5	13.8 ± 2.9	9.21 ± 1.6	14.2 ± 1.5

Data given are mean ± SD

*CONT* the control group received Cu in 100 % of daily demand from sulfate, *OG100* the group received Cu in 100 % of daily demand from Cu glycine, *OG75* the group received Cu in 75 % of daily demand from Cu glycine, *OG50* the group received Cu in 50 % of daily demand from Cu glycine

Differences between superscript letters means significant differences with *P* < 0.05

The Cu supplementation in the organic form did not influence the histomorphometric parameters of the nerve plexus in the jejunum compared to the Cu supplementation in the inorganic form (Table 4).

**Table 4:** The histomorphometry of nerve plexuses in jejunum of control rats and supplemented with different level of dietary Cu

	CONT	OG100	OG75	OG50
Auerbach plexus				
Area [μm^2^]	578 ± 529	700 ± 622	617 ± 671	678 ± 715
Perimeter [μm]	133 ± 89	126 ± 75	123 ± 113	133 ± 102
Mean Feret [μm]	40.5 ± 26.4	38.3 ± 22.4	37.1 ± 33.7	39.9 ± 30.5
Min diameter [μm]	11.5 ± 4.6	14.4 ± 5.6	13.7 ± 5.5	13.5 ± 4.6
Mean diameter [μm]	21.3 ± 7.9	24.5 ± 9.5	22.3 ± 8.9	22 ± 8.6
Sphericity	0.11 ± 0.13	0.18 ± 0.15	0.24 ± 0.22	0.22 ± 0.21
Meissner plexus				
Area [μm^2^]	263 ± 172	321 ± 287	182 ± 167	360 ± 264
Perimeter [μm]	68 ± 27	80 ± 43	55 ± 30	82 ± 42
Mean Feret [μm]	20.2 ± 7.7	23.6 ± 12.0	16.3 ± 8.9	24.4 ± 12
Min diameter [μm]	12.1 ± 3.6	11.2 ± 3.9	9.9 ± 3.8	13.1 ± 3.9
Mean diameter [μm]	16.7 ± 4.9	17.5 ± 5.9	13.3 ± 5.4	19.0 ± 5.9
Sphericity	0.41 ± 0.24	0.22 ± 0.16	0.42 ± 0.0.5	0.31 ± 0.18

Data given are mean ± SD

*CONT* the control group received Cu in 100 % of daily demand from sulfate, *OG100* the group received Cu in 100 % of daily demand from Cu glycine, *OG75* the group received Cu in 75 % of daily demand from Cu glycine, *OG50* the group received Cu in 50 % of daily demand from Cu glycine

## Discussion

Cu deficiency can occur early in life when elevated requirements are evident during rapid growth. For the first time, the full spectrum of Cu deficiency was shown in 1964 when Cu was considered the cause of anemia. Then, infants and children receiving copper-free total parenteral nutrition served to clearly define copper as an essential nutrient for human infants. Further, tissue Cu deficiency as a result of an inherited defect of Cu transport was also described as Menkes-Syndrom [18]. On the other hand, Cu accumulation in the liver is observed in another inherited disorder related to Cu metabolism, namely Wilson’s disease with a simultaneously decreased Cu serum concentration [3]. However, sometimes, dietary supplements containing Cu are the main sources of this trace element. Thus, it is necessary to examine the effects of the supplementation of Cu on intestine histomorphometric changes in conditions of full coverage of the daily requirement or low, deficient intake of Cu in relation to the daily requirement defined as providing a minimum of given trace element in order to cover a required amount to maintain normal homeostasis for a 24-h period in rats. In the current study, two of the diets supplemented with organic Cu as Cu-Gly covered the daily demand in 75 and 50 %.

The obtained results demonstrated that there was no direct effect of the dietary Cu-glycine complex in 100, 75, and 50 % of daily demand on feed intake in adolescent rats. Additionally, Cu supplementation at the experimentally lowered level in Cu-glycine complex did not affect the body weight. This is in agreement with an earlier study also performed on adolescent rats supplemented with Cu as chelate at the level of 25, 50, and 75 % of the required daily amount [15]. However, this result differed from others reporting that Cu deficiency in relation to daily requirement leads to a reduced body mass because Cu is essential for normal growth and development [18–20].

The current study indicated that supplementation with Cu did not influence biochemical and basal hematologicalal parameters in the adolescent rats (irrespective of the Cu amount in the diet). It is worth mentioning that Cu-deficient diet does not affect the final body mass of adult rats. Moreover, in our adolescent animals, there was no difference in the Cu plasma concentration between the control group supplemented with the Cu sulfate in 100 % daily demand and the groups supplemented with Cu-glycine complex at the level below the daily demand for the rat. This may indicate that there was sufficient Cu absorption from the intestine in adolescent rats. A similar study showed that in rats fed with Cu amino acid chelate lowered to 50 %, and even to 25 % of daily demand, there was no Cu liver deficiency [15]. Moreover, numerous studies in animals and human volunteers show a link between Cu deficiency and altered lipid metabolism [21–24]. An increased concentration of total cholesterol and LDL cholesterol and a reduction of HDL cholesterol were observed in subjects fed an experimental Cu-low diet because of more rapid synthesis and clearance thereof into blood plasma and a limited cholesterol pool for excretion as biliary steroids [25]. A low Cu intake was also shown to diminish glucose tolerance [18].

Our study performed on adolescent rats showed that Cu given in the diet in Cu-glycine complex irrespective of its dose did not alter glucose and lipid metabolism. The lack of differences in the glucose concentration between the investigated groups can indicate that our adolescent rats were not characterized by lowered activity of enzymes responsible for metabolic transformation of glycogen in the body and abnormal accumulation thereof, i.e., glycogenosis.

Copper homeostasis and status are regulated at the whole body level mainly by intestinal absorption. Copper supplementation in the broiler diet at a dose higher than 250 mg/kg depressed the height of villi and thickened the muscular layer in the duodenum [26]. Study performed on weanling pigs shows that duodenal villus height was reduced in the group supplemented with Cu sulfate, but inorganic salt was given in higher amount (225 mg of Cu/kg of a diet) than the minimum daily dose by American standards that recommended 6–125 ppm depending on production cycle [27, 28]. A different Cu study showed that rats fed with diet supplemented with CuSO_4_ at the dose of 80 mg/kg body weight had no effects on the villus height and crypt depth of small intestinal mucosa [29]. However, Cu-deficient diet (less than 1 mg Cu/kg) in cattle resulted in lesions of the small intestine [30]. The morphology of villi changes depending on the examined segment of the intestine and its function, age, and diet, and environmental factors. Rat studies showed that Cu deficiency affected the enteric nervous system without significant changes in secretory function [31].

Our study showed that the Cu-deficient diet in rats did not affect the enteric nervous system and probably did not influence its function (Table 4). Nevertheless, on the basis of the histomorphometric analysis of the jejunum, the current study indicated that Cu given in the organic form covering 100 % of daily demand in adolescent rats depressed the number and height of enterocytes (Table 3). In turn, organic Cu given in the amount lowered to 75 or 50 % of daily demand did not influence the morphology of enterocytes. Changes in the morphology of enterocytes, however, can have a decisive influence on their function related to contact digestion within the brush-border membrane and absorption of the end products of digestion. Moreover, a tendency to a decreased number and height of villi was observed, especially in the groups supplemented with the organic form of Cu (in the amount of 100 and 75 % of daily demand). Even slight changes in the shape or length of the villi substantially affect the absorptive area. It should be noted that the growth of a young villus is an effect of decreased exfoliation of apoptotic cells at the top of the villi but not increased mitotic activity in the zone of renewal in the intestinal crypts. Probably, when enterocytes move from the renewal zone toward the top of the villus, epithelial cells mature, differentiate rapidly, and undergo apoptosis leading to villus shortening when organic Cu is present in the diet in the amount of 100 or 75 % of daily demand. Despite the increased proliferation and migration of enterocytes, the renewal of damaged and massively exfoliated epithelium is insufficient and might lead to flattening and villar atrophy. Our rats fed with organic Cu showed a tendency to reduction of the number of villi. On the other hand, this might be caused by the decline in the renewal process as an effect of the decrease in the number of active crypts and the increase in the number of inactive crypt in rats fed with diet containing organic Cu in the amount of 100 % of daily demand.

## Conclusions

Cu given to adolescent rats in the diet in the organic form covering an amount of the full daily demand in 100 % appears to be more harmful with regard to the intestinal epithelium than when administered in 75 or 50 % of daily demand and compared to the inorganic dietary Cu supplementation in the amount of 100 % of daily demand. Organic dietary Cu supplementation probably influenced the villar developmental process and led to massive cell exfoliation on the upper half of the villus tip, thereby shortening villar length and the number of enterocytes. The damage of jejuna epithelium can cause the disease and infection in the intestinal tract, the development of pathogenic intestinal microflora, and decreased immunity, which can lead to the development of food allergies. From the point of view of the digestive function of the intestinal epithelium, it is beneficial to reduce the daily dose of organic Cu as a dietary supplement.
